# Maternal health and birth outcomes in a South African birth cohort study

**DOI:** 10.1371/journal.pone.0222399

**Published:** 2019-11-21

**Authors:** Heather J. Zar, Jennifer A. Pellowski, Sophie Cohen, Whitney Barnett, Aneesa Vanker, Nastassja Koen, Dan J. Stein

**Affiliations:** 1 Department of Paediatrics and Child Health, Red Cross War Memorial Children’s Hospital and SA-MRC Unit on Child & Adolescent Health, University of Cape Town, Cape Town, South Africa; 2 Department of Behavioral and Social Sciences, School of Public Health, Brown University, Providence, Rhode Island, United States of America; 3 Department of Psychiatry and Mental Health and SA-MRC Unit on Risk and Resilience in Mental Disorders, University of Cape Town, Cape Town, South Africa; Monash University, AUSTRALIA

## Abstract

**Background:**

Maternal physical and mental health during pregnancy are key determinants of birth outcomes. There are relatively few prospective data that integrate physical and mental maternal health measures with birth outcomes in low- and middle-income country settings. We aimed to investigate maternal health during pregnancy and the impact on birth outcomes in an African birth cohort study, the Drakenstein Child Health Study.

**Methods:**

Pregnant women attending 2 public health clinics, Mbekweni (serving a predominantly black African population) and TC Newman (predominantly mixed ancestry) in a poor peri-urban area of South Africa were enrolled in their second trimester and followed through childbirth. All births occurred at a single public hospital. Maternal sociodemographic, physical and psychosocial characteristics were comprehensively assessed. Multivariable linear regression models were used to explore associations between maternal health and birth outcomes.

**Results:**

Over 3 years, 1137 women (median age 25.8 years; 21% HIV-infected) gave birth to 1143 live babies. Most pregnancies were uncomplicated but gestational diabetes (1%), anaemia (22%) or pre-eclampsia (2%) occurred in a minority. Most households (87%) had a monthly income of less than USD 350; only 27% of moms were employed and food insecurity was common (37%). Most babies (80%) were born by vaginal delivery at full term; 17% were preterm, predominantly late preterm. Only 74 (7%) of babies required hospitalisation immediately after birth and only 2 babies were HIV-infected. Food insecurity, socioeconomic status, pregnancy-associated hypertension, pre-eclampsia, gestational diabetes and mixed ancestry were associated with lower infant gestational age while maternal BMI at enrolment was associated with higher infant gestational age. Primigravida or alcohol use during pregnancy were negatively associated with infant birth weight and head circumference. Maternal BMI at enrolment was positively associated with birth weight and gestational diabetes was positively associated with birth weight and head circumference for gestational age. Smoking during pregnancy was associated with lower infant birth weight.

**Conclusion:**

Several modifiable risk factors including food insecurity, smoking, and alcohol consumption during pregnancy were identified as associated with negative birth outcomes, all of which are amenable to public health interventions. Interventions to address key exposures influencing birth outcomes are needed to improve maternal and child health in low-middle income country settings.

## Introduction

Maternal health during pregnancy is a key determinant of birth outcomes and child health, with accumulating evidence that the roots of chronic illness may be influenced by prenatal exposures.[1–3] Optimizing maternal physical and mental health and reducing potentially harmful exposures during pregnancy are therefore key to promoting child health and to reducing chronic illness. Few studies have, however, prospectively or comprehensively investigated the impact of physical and mental health during pregnancy on birth outcomes and subsequent infant health. Further, studies have predominantly been done in high-income countries, but the population of women of childbearing age is greatest in low and middle income countries (LMICs).[4]

In LMIC settings, pregnant women may have particularly high intensity exposure to many potentially harmful risk factors to their own health and to that of their child such as HIV, hazardous alcohol use, or tobacco smoke exposure.[5, 6] Poverty associated environmental factors such as poor housing or crowded living conditions may exacerbate exposure risk.[7] Maternal malnutrition has been linked to a number of poor fetal and birth outcomes including neural tube defects, preterm delivery, and low birth weight.[8, 9] Food insecurity not only reflects inaccessibility to food but also encapsulates the psychological impact of worrying about having enough food. Less research has focused on the influence of food insecurity on birth outcomes, particularly in LMIC settings.[10] Given the relationship between chronic stress and poor birth outcomes, such as low birth weight[11], food insecurity should be further investigated as a predictor of poor birth outcomes.

Physical illness, such as hypertension has been associated with adverse birth outcomes[12] and may be relatively easily identified on antenatal screening. In contrast mental disorders may be very common in LMIC, but are less frequently recognised or treated in such settings, and their association with birth outcomes and child health has not been well studied in this context.[13] Further complicating our understanding of the influences maternal physical and mental health on fetal health and birth outcomes, is their interactive relationships with sociodemographic and nutritional factors.[14] For example, food insecurity has been associated with mental health issues among mothers, particularly depression and alcohol use.[15] Given the interactive nature of nutrition, physical health, and mental health for pregnant women, it is imperative to consider these factors in tandem when predicting birth outcomes.[16] Models that include sociodemographic, food insecurity, physical health, mental health, and substance use predictors of birth outcomes are needed to disentangle impacts on birth outcomes and to guide intervention efforts to improve maternal and infant health in LMICs.[17]

The aim of this study was to comprehensively investigate maternal physical and mental health during pregnancy and the impact on birth outcomes in a South African birth cohort study, the Drakenstein Child Health Study (DCHS).[18] This study is unique given its location in a poor African community and the ability to undertake comprehensive measures of maternal physical and mental health factors longitudinally through the antenatal period. We prospectively investigated maternal sociodemographic, food insecurity, physical health, mental health, and substance use factors and the association of these with infant birth outcomes.

## Methods

The DCHS is a multidisciplinary population-based birth cohort study situated in the Drakenstein area in Paarl, a peri-urban area, 60km outside Cape Town, South Africa.[18] The population comprise approximately 200 000 people with little immigration or emigration. The public health system includes well-established primary health clinics providing antenatal care and HIV treatment and prevention programs including prevention of mother to child transmission (PMTCT). All births occur at a single hospital, Paarl Hospital. More than 90% of the population access health care in the public sector including antenatal services. Maternal physical and mental health was investigated through longitudinal measurements through pregnancy and birth, as were socio-demographic factors and psychosocial risk factors.[18–20]

### Participants

Consenting pregnant women were enrolled from March 2012 to March 2015 and followed through childbirth. Women were enrolled in their second trimester (20–28 weeks gestation) at 2 public sector primary health care clinics, one serving a predominantly mixed ancestry population (TC Newman) and the other serving a predominantly Black African population (Mbekweni). As per the national health program, antenatal and obstetric care was provided free to women in these health care facilities. Women were eligible to participate if they attended one of the two study clinics, were at least 18 years of age and intended to remain resident in the study area for at least 1 year. All assessments were available in English, isiXhosa, and Afrikaans and participants chose their preferred language.

### Measures

#### Sociodemographics and food insecurity

Sociodemographic variables including age, marital status, employment, and income were measured through questionnaires and antenatal visits. Socioeconomic status (SES) was measured based on a composite score of asset ownership, household income, employment and education, adapted from items used in the South African Stress and Health Study (SASH).[13] Perceived household food insecurity was assessed using an adapted version of the short form of the USDA Household Food Security Scale[21] (detailed in Pellowski et al.).[22]

#### Physical health

Maternal physical health was assessed at enrolment, at a follow-up antenatal visit and at birth through questionnaires and physical examination conducted by trained study staff on study-specific equipment. Maternal blood pressure (single arm, single measurement using an electronic blood pressure cuff) and weight were monitored antenatally. All pregnancy complications were collected prospectively (at enrolment and a second antenatal visit) as well as from chart review. High blood pressure was defined as having a BP ≥140/90 mmHg. Pre-eclampsia was defined as new onset of hypertension after 20 weeks gestation with proteinuria or other organ dysfunction. Eclampsia was defined as the presence of seizures due to pre-eclampsia. Haemoglobin measurement was done in the antenatal period as part of routine care. Anaemia was calculated conservatively as any haemoglobin measurement <10 g/dl. WHO guidelines define moderate or severe anaemia as <10 g/dl. Gestational diabetes was assessed through urine dipstick and fasting blood glucose if urine glucose was positive. No formal glucose tolerance tests were conducted. Routine antenatal care included HIV rapid testing on enrolment if a mother’s HIV status was unknown.[23] Syphilis serology, hemoglobin measurement and urine dipstick analysis for proteinuria or white cells were performed; urine analysis was repeated through pregnancy.

#### Maternal mental health

Maternal mental health was measured using validated questionnaires administered by trained study staff at an antenatal visit at 28 to 32 weeks’ gestation. The Edinburgh Postnatal Depression Scale (EPDS) was used to assess symptoms of depression and has been validated for use in both postpartum and pregnant women. The EPDS comprises 10 items, each scored on a severity scale ranging from 0 to 3. A total score of 13 or greater indicates probable depression. The SRQ-20 is a WHO-endorsed measure of psychological distress; a cut-off score of ≥8 was used to dichotomize participants into high and low risk categories.[24] [25]The Intimate Partner Violence (IPV) Questionnaire used in this study was adapted from the WHO multi-country study and the Women’s Health Study in Zimbabwe.[26] Participants were dichotomized into exposed or unexposed for having experienced emotional, physical or sexual IPV in the past 12 months.

#### Substance use during pregnancy

The Alcohol, Smoking and Substance Involvement Screening Test (ASSIST)[27] was used to assess self-report of tobacco, alcohol, and other substance use (during the past three months). Maternal smoking or passive smoke exposure was measured by urine cotinine antenatally and at birth using the IMMULITE^R^ 1000 Nicotine Metabolite Kit (Siemens Medical Solutions Diagnostics^R^, Glyn Rhonwy, United Kingdom) to distinguish categories of smoke exposure i.e. levels <10 ng/ml (non-smoker), 10–499 ng/ml (passive smoke exposure) or ≥500 ng/ml (active smoker).

#### Pregnancy and birth outcomes

Pregnancy data were collected from mothers at two antenatal visits. Study staff attended all deliveries, and recorded mode of delivery, development of any complications and infant birth outcomes. Mothers and infants were followed until discharge from Paarl hospital. Birth weight, length and head circumference were measured at birth by trained staff. Gestational age at birth was estimated based on an antenatal ultrasound done in the second trimester; if this was unavailable then symphysis-fundal height, recorded by trained clinical staff at enrolment, or maternal recall of last menstrual period was used.

### Ethics

Ethical approval was obtained from the Faculty of Health Sciences Research Ethics Committee, University of Cape Town (401/2009) and the Provincial Research committee. Mothers gave written informed consent at enrolment.

### Data analysis

Data were analysed using Stata 12 (StataCorp Inc, College Station, Texas, USA). Demographics, physical and mental health, pregnancy- and birth-related data were described using median (interquartile range (IQR)) or number (%). Outliers were not deleted. Data were compared between black African and mixed ancestry participants using the Mann-Whitney *U* test and the χ^2^ test. Birth weight, length and head circumference were converted to Z-scores for gender and gestational age using the revised 2003 Fenton curves which harmonizes the preterm growth charts with the new WHO growth standards.[28] Predictors of poor birth outcomes (gestational age at delivery in weeks; birthweight and head circumference Z-scores) were identified using multivariable linear regression models. The basic multivariable model included maternal age, marital status, SES and food security. For additional covariates, univariable analyses were conducted. Significant univariable coefficients (p<0.15) were included in multivariable hierarchical regression models. In multivariable hierarchical regression analyses, a *P*-value<0.05 was considered statistically significant. Data will be made available on request.

## Results

Of the 1225 participants enrolled in this birth cohort there were 1143 known live infants born to 1137 mothers, including 4 sets of twins and 1 set of triplets. There were 13 (1.1%) stillbirths (two were a pair of twins), 9 (0.7%) miscarriages, and 67 (5.5%) mothers were lost to follow-up prior to delivery.

### Sociodemographics

Mean estimated gestational age at enrolment was 23.4 weeks (SD = 3.4). The study population (55% Black African; median age 25.8 years (IQR 22.0–30.8)) was predominantly of low SES, with only 27% of mothers currently employed and 38% reported a household income less than R1000 (approximately 71 USD) per month (Table 1). Almost half (48%) of all women were receiving national social assistance. Only 39% had completed secondary (high school) education or higher. Less than half (40%) reported having a stable partner. Food insecurity was common occurring in 37% of homes. Compared to participants of mixed ancestry, black African women were older, had a lower household income, achieved a lower level of education and had more food insecurity, Table 1.

**Table 1:** Demographic, physical health and psychosocial characteristics of pregnant mothers enrolled in the birth cohort study (N = 1137).

Variable		N	Black African	Mixed ancestry	Total	*P*-value
			(Mbekweni)	(TC Newman)		
Number of mothers			628 (55)	509 (45)	1137 (100)	
Median age at enrolment (years)		1137	26.7 (22.3–31.6)	24.7 (21.4–29.2)	25.8 (22.0–30.8)	<0.001***
Currently employed		1137	157 (25)	149 (29)	306 (27)	0.106
Currently married or cohabiting		1136	237 (38)	221 (43)	458 (40)	0.105
Receiving social assistance (government grant)		1135	306 (49)	242 (48)	548 (48)	0.739
Highest Education Level attained						
	Primary	1137	49 (8)	37 (7)	86 (8)	0.038*
	Some secondary		340 (54)	266 (52)	606 (53)	
	Completed secondary		189 (30)	183 (36)	372 (33)	
	Any tertiary		50 (8)	23 (5)	73 (6)	
Average household income per month						
	<1000ZAR (71 USD)	1137	263 (42)	167 (33)	430 (38)	<0.001***
	1000-5000ZAR (71–356 USD)		299 (48)	254 (50)	553 (49)	
	>5000ZAR (>356 USD)		66 (10)	88 (17)	154 (14)	
SES Quartiles						
	Lowest SES	1137	192 (31)	88 (17)	280 (25)	<0.001***
	Low-moderate SES		157 (25)	133 (26)	290 (25)	
	Moderate-high SES		154 (25)	136 (27)	290 (25)	
	High SES		125 (20)	152 (30)	277 (24)	
Food Insecure		1014	295 (53)	83 (18)	378 (37)	<0.001***
**Physical health**						
Weight at enrolment (kg)		1129	72 (61–85)	60 (52–73)	67 (57–81)	<0.001***
Height at enrolment (m)		1122	1.60 (1.56–1.65)	1.58 (1.54–1.62)	1.59 (1.55–1.64)	<0.001***
BMI at enrolment (kg/m2)		1117	28 (24–33)	24 (21–29)	26 (22–32)	<0.001***
HIV-infected		1137	227 (36)	16 (3)	243 (21)	<0.001***
	On ART	238	220 (99)	16 (100)	236 (99)	0.703
	Median CD4 Count (cells/uL)	205	408 (284–594)	556 (347–677)	411 (286–609)	0.511
	Undetectable Viral Load (<40 copies/mL)	148	98 (72)	3 (50)	101 (70)	0.257
**Pregnancy**						
Primigravida		1133	202 (32)	197 (39)	399 (35)	0.022*
Planned Pregnancy		1134	176 (28)	173 (34)	349 (31)	0.031*
Pregnancy complications						
	Hypertension	1130	30 (5)	22 (4)	52 (5)	0.685
	Eclampsia	1130	1 (0)	2 (0)	3 (0)	0.451
	Pre-eclampsia	1130	13 (2)	14 (3)	27 (2)	0.472
	Anemia	1076	149 (25)	85 (17)	234 (22)	0.002**
	Gestational Diabetes	1129	6 (1)	7 (1)	13 (1)	0.523
	Asthma	1130	6 (1)	9 (2)	15 (1)	0.241
Lab test during pregnancy						
	Positive RPR	1046	15 (2)	12 (3)	27 (3)	0.745
	Positive VDRL	222	3 (7)	8 (4)	11 (5)	0.468
	Hemoglobin (g/dL)	1065	11.1 (10.2–12.1)	11.4 (10.4–12.5)	11.2 (10.3–12.3)	0.055
**Psychosocial and Substance Use**						
EPDS (above threshold)		994	124 (23)	113 (25)	237 (24)	0.478
SRQ-20 (above threshold)		994	92 (17)	110 (24)	202 (20)	0.005**
IPV (above threshold)						
	Emotional	994	108 (20)	157 (35)	265 (27)	<0.001***
	Physical	994	110 (20)	107 (24)	217 (22)	0.237
	Sexual	994	19 (4)	49 (11)	68 (7)	<0.001***
Alcohol use risk						
	No use	992	486 (91)	126 (28)	612 (62)	<0.001***
	Lower risk		15 (3)	259 (57)	274 (28)	
	Moderate risk		12 (2)	65 (14)	77 (8)	
	High risk		24 (4)	5 (1)	29 (3)	
Cotinine						
	Non-smoker	1069	193 (33)	55 (11)	248 (23)	<0.001***
	Passive smoker		299 (51)	179 (37)	478 (45)	
	Active smoker		89 (15)	254 (52)	343 (32)	

Notes: Medians (IQR ranges) are reported for maternal age, weight, height and BMI at enrolment, CD4 count, and hemoglobin. All other results show frequencies (percentages). SES = socioeconomic status; BMI = body mass index; ART = antiretroviral therapy; RPR = rapid plasma reagin test; VDRL = venereal disease research laboratory; EPDS = Edinburgh Postnatal Depression Scale (validated in pregnancy); SRQ-20 = self-reporting questionnaire 20-item; IPV = intimate partner violence

*p<0.05

**p<0.01

***p<0.001

### Maternal physical health and pregnancy complications

Most participants (65%) had previously been pregnant, and the majority of current pregnancies (69%) were unplanned (Table 1). Gestational complications developed in a minority of women, including gestational diabetes in 13 (1%), anemia in 234 (22%), hypertension in 52 (5%), pre-eclampsia in 27 (2%), and asthma in 15 (1%). There were 243 (21%) HIV-infected mothers, Table 1. Of the HIV-infected mothers, nearly all were on ART during pregnancy (99%), were relatively healthy with a median CD4 T-cell count of 411 (IQR 286–609), and most had an undetectable viral load (<40 copies/mL; 70%). Compared to participants of mixed ancestry, black African women had higher BMIs at enrolment, had a higher prevalence of HIV infection, were less likely to be first time mothers, were less likely to have planned their pregnancy, and were more likely to have anaemia, Table 1.

### Psychosocial factors and substance use

Mental health issues were common including probable depression (EPDS above threshold: 24%) and psychological distress (SRQ-20 above threshold: 20%, (Table 1). Exposure to violence was also high with 27% reporting emotional IPV in the past year, 22% reporting physical IPV, 7% reporting sexual IPV. Self-reported current alcohol use risk was high in 29 (3%), moderate in 77 (8%) and present but lower in 274 (28%) of pregnant women. Urine cotinine results indicated that 343 (32%) were active smokers, 478 (45%) had passive smoke exposure, and 248 (23%) were non-smokers. Mental health and substance use differed by community, with higher rates amongst mixed ancestry women on psychological distress, emotional IPV, sexual IPV, alcohol use risk, and actively smoking.

### Birth outcomes

Most births (n = 901, 80%) were by normal vaginal delivery. There were 148 (13%) acute Caesarean and 76 (7%) elective Caesarean deliveries (Table 2). The median gestational age was 39 (37–40) weeks and 191 (17%) infants were born preterm. Amongst these, most were late preterm births, with a median gestational age of 35 (32–36) weeks. Most babies were appropriate size, with a median WAZ score -0.6 SD (-1.3 to 0.1), HAZ score 0 SD (-0.9 to 0.9) and median head circumference Z-score -0.5 SD (-1.3 to 0.3). Low birth weight for gestational age (<-2SD) occurred in 99 infants (9%). No baby had any observable gross deformity at birth. Of 244 HIV-exposed infants, only 2 (1%) were known to be HIV-infected; they tested positive postnatally (age 2 and 3.7 months). Most mother-infant pairs were discharged home soon after birth but 74 (7%) were hospitalised after birth of which 71 (6%) had respiratory difficulties including 20 (2%) requiring oxygen support.

**Table 2:** Birth outcomes of the 1143 births including 4 sets of twins and 1 set of triplets.

Variable		N	Black African	Mixed ancestry	Total	*P*-value
			(Mbekweni)	(TC Newman)		
**Delivery**						
Delivery method	Vaginal	1133	480 (77)	424 (83)	904 (80)	0.011*
	Caesarean Elective		54 (9)	25 (5)	79 (7)	
	Caesarean Acute		91 (15)	59 (12)	150 (13)	
Anaesthetics use during labour						
	Epidural	206	3 (2)	2 (2)	5 (2)	0.831
	Spinal		114 (91)	72 (89)	186 (90)	
	General		8 (6)	7 (9)	15 (7)	
**Birth**						
Gender	Girl	1142	318 (50)	232 (46)	550 (48)	0.117
	Boy		315 (50)	277 (54)	592 (52)	
Multiple births	Singletons	1132	618 (98)	509 (100)	1127 (99)	0.028*
	Twins		4 (1)	0 (0)	4 (0)	
	Triplets		1 (0)	0 (0)	1 (0)	
Gestational age (weeks)		1137	39 (38 to 40)	39 (37 to 40)	39 (37 to 40)	0.214
	Term (> = 37 weeks)		520 (83)	426 (84)	946 (83)	0.689
	Preterm (<37 weeks)		108 (17)	83 (16)	191 (17)	
Birth Weight (g)		1127	3150 (2780 to 3450)	3000 (2630 to 3350)	3080 (2710 to 3420)	<0.001***
Birth Weight-for-GA			-0.4 (-1.2 to 0.2)	-0.7 (-1.4 to -0.1)	-0.6 (-1.3 to 0.1)	<0.01**
	<-2SD		48 (8)	51 (10)	99 (9)	0.166
Birth Length (cm)		1107	50 (48 to 52)	50 (47 to 52)	50 (48 to 52)	0.011*
Birth Length-for-GA			0.1 (-0.9 to 1.0)	0.0 (-0.9 to 0.8)	0.0 (-0.9 to 0.9)	0.167
	<-2SD		49 (8)	52 (10)	101 (9)	0.181
Head Circumference (cm)		1119	34 (33 to 35)	34 (32 to 35)	34 (32 to 35)	<0.001***
Head Circumference-for-GA			-0.4 (-1.2 to 0.4)	-0.6 (-1.4 to 0.2)	-0.5 (-1.3 to 0.3)	0.014*
	<-2SD		67 (11)	63 (13)	130 (12)	0.380
Respiratory difficulty		1128	41 (7)	30 (6)	71 (6)	0.626
	Requiring oxygen	1116	15 (2)	5 (1)	20 (2)	0.092
Jaundiced		1129	24 (4)	31 (6)	55 (5)	0.145
Low blood glucose (<3 mmol/L)		1112	199 (33)	179 (36)	378 (34)	0.264
Known Infant HIV Infection		209	2 (1)	0 (0)	2 (1)	0.726

*p<0.05

**p<0.01

***p<0.001

#### Maternal predictors of gestational age

In the final model (Block 4, Table 3), several demographic variables significantly predicted gestational age: mothers who were food insecure (B = -0.542, p = 0.003) were significantly more likely to have infants with lower gestational age. Mothers who had moderate-high or high SES (B = 0.543, p = 0.019; B = 0.605, p = 0.014, respectively) had infants with higher gestational ages. Mothers who had pregnancy related hypertension (B = -1.226, p = 0.004), pre-eclampsia or eclampsia (B = -1.741, p = 0.001) or gestational diabetes (B = -2.837, p = 0.001) were more likely to have infants with lower gestational ages. Mothers who had anemia were significantly more likely to have infants with higher gestational ages (B = 0.420, p = 0.031). Mothers with higher BMIs at enrolment were significantly more likely to have infants with higher gestational age (B = 0.078, p<0.001) as were first time mothers (B = 0.481, p = 0.025).

**Table 3:** Hierarchical regression predicting gestational age (N Final model = 930).

		Block 1			Block 2			Block 3^			Block 4		
Variables		Coefficient	95% CI LL	95% CI UL	Coefficient	95% CI LL	95% CI UL	Coefficient	95% CI LL	95% CI UL	Coefficient	95% CI LL	95% CI UL
Demographics													
Clinic													
	Mbekweni	-	-	-	-	-	-	-	-	-	-	-	-
	TC Newman	-0.517**	-0.865	-0.170	-0.293	-0.677	0.091	-0.293	-0.677	0.091	-0.231	-0.654	0.192
Maternal age		-0.016	-0.046	0.014	-0.028	-0.060	0.005	-0.028	-0.060	0.005	-0.006	-0.042	0.030
Married or cohabitating		0.147	-0.200	0.495	0.067	-0.284	0.418	0.067	-0.284	0.418	0.103	-0.254	0.459
SES													
	Low	-	-	-	-	-	-	-	-	-	-	-	-
	Low-moderate	0.246	-0.198	0.690	0.174	-0.268	0.616	0.174	-0.268	0.616	0.227	-0.214	0.668
	Moderate-high	0.707**	0.257	1.157	0.660**	0.209	1.111	0.660**	0.209	1.111	0.543**	0.090	0.996
	High	0.782**	0.311	1.254	0.753**	0.277	1.229	0.753**	0.277	1.229	0.605**	0.120	1.089
Food Insecure		-0.598**	-0.954	-0.242		-0.562**	-0.924	-0.200		-0.562**	-0.924	-0.200		-0.542**	-0.904	-0.179
Pregnancy and Physical Health													
BMI at Enrollment					0.073***	0.045	0.100	0.073***	0.045	0.100	0.078***	0.050	0.106
Caesarean (Elective or Acute)					0.180	-0.228	0.587	0.180	-0.228	0.587	0.133	-0.271	0.537
Pregnancy Complications					-	-	-	-	-	-	-	-	-
	Hypertension				-1.245**	-2.068	-0.422	-1.245**	-2.068	-0.422	-1.226**	0.050	0.106
	(Pre)-eclampsia				-2.147***	-3.180	-1.113	-2.147***	-3.180	-1.113	-1.741**	-0.271	0.537
	Anaemia				0.456**	0.069	0.842	0.456**	0.069	0.842	0.420*	0.050	0.106
	Gestational diabetes				-2.745**	-4.401	-1.090	-2.745**	-4.401	-1.090	-2.837**	-0.271	0.537
	Asthma				-	-	-	-	-	-	-	-	-
HIV Infected					-0.372	-0.810	0.067	-0.372	-0.810	0.067	-0.368	0.050	0.106
Mental Health and Psychosocial Factors													
Depressive Symptoms (EPDS score)								-	-	-	-	-	-
Psychological Distress (SRQ-20)								-	-	-	-	-	-
IPV during pregnancy								-	-	-	-	-	-
Substance Use During Pregnancy & Primigravida													
Alcohol use (moderate-high risk)											-	-	-
Cotinine level													
	Non-smoker										-	-	-
	Passive										0.015	-0.400	0.430
	Active										-0.213	-0.704	0.279
Primigravida											0.481*	0.061	0.901
			R2	p-value		R2	p-value		R2	p-value		R2	p-value
			0.033	<0.001		0.108	<0.001		-	-		0.111	<0.001
						Change R2	p-value		Change R2	p-value		Change R2	p-value
						0.075	<0.001		-	-		0.003	1.000

Note: ^No mental health or psychosocial factors significantly predicted gestational age in bivariate analyses, thus, no new variables were added to this block.

*p<0.05

**p<0.01

***p<0.001; 95% CI LL = 95% Confidence Interval Lower Limit, 95% CI UL = 95% Confidence Interval Upper Limit

In this hierarchical regression, the demographic variable block (R^2^ = 0.033, p<0.001) and the pregnancy and physical health block (change in R^2^ = 0.760, p<0.001) significantly contributed to the explained variance in the final model but the substance use variable block did not (Table 3).

### Maternal predictors of birth weight adjusted for gestational age

In the final model (Block 4, Table 4), no demographic variables significantly predicted birth weight adjusted for gestational age when controlling for other variables. Mothers with higher BMIs at enrollment were significantly more likely to have infants with higher adjusted birth weights (B = 0.015, p = 0.014). First time mothers were more likely to have infants with lower adjusted birth weights (B = -0.337, p<0.001). Mothers who used alcohol (B = -0.351, p = 0.002) and mothers who were active smokers (B = -0.273, p = 0.013) were more likely to have infants with lower adjusted birth weights. In this hierarchical regression, the demographic variable block (R^2^ = 0.024, p = 0.001), the pregnancy and physical health block (change in R^2^ = 0.027, p = 0.001), the mental health block (change in R^2^ = 0.010, p = 0.046), and the substance use block (change in R^2^ = 0.035 p<0.001) all significantly contributed to the explained variance in the final model (Table 4).

**Table 4:** Hierarchical regression predicting birth weight for gestational age (Z-score; N Final model = 896).

		Block 1			Block 2			Block 3			Block 4		
Variables		Coefficient	95% CI LL	95% CI UL	Coefficient	95% CI LL	95% CI UL	Coefficient	95% CI LL	95% CI UL	Coefficient	95% CI LL	95% CI UL
Demographics													
Clinic													
	Mbekweni	-	-	-	-	-	-	-	-	-	-	-	-
	TC Newman	-0.259**	-0.406	-0.111	-0.195*	-0.361	-0.028	-0.144	-0.314	0.026	-0.010	-0.196	0.176
Maternal age		0.010	-0.002	0.023	0.003	-0.011	0.017	0.009	-0.005	0.023	-0.004	-0.019	0.012
Married or cohabitating		0.039	-0.108	0.186	0.064	-0.087	0.215	0.089	-0.062	0.239	0.021	-0.132	0.175
SES													
	Low	-	-	-	-	-	-	-	-	-	-	-	-
	Low-moderate	-0.061	-0.249	0.128	-0.094	-0.285	0.097	-0.082	-0.272	0.108	-0.059	-0.249	0.131
	Moderate-high	-0.041	-0.231	0.149	-0.064	-0.257	0.130	-0.056	-0.249	0.138	-0.028	-0.222	0.167
	High	0.178	-0.022	0.378	0.131	-0.074	0.336	0.115	-0.089	0.320	0.109	-0.099	0.318
Food Insecure		-0.036	-0.187	0.115	0.057	-0.099	0.213	0.078	-0.081	0.236	0.078	-0.080	0.236
Pregnancy and Physical Health													
BMI at Enrollment					0.024***	0.012	0.036	0.020**	0.008	0.032	0.015*	0.003	0.027
Pregnancy Complications													
	Hypertension				-0.111	-0.474	0.253	-0.093	-0.455	0.268	-0.207	-0.579	0.165
	(Pre)-eclampsia				-0.329	-0.785	0.126	-0.329	-0.776	0.119	-0.263	-0.708	0.182
	Anaemia				0.051	-0.116	0.217	0.051	-0.115	0.217	0.044	-0.120	0.208
	Gestational diabetes				0.394	-0.319	1.107	0.622	-0.120	1.363	0.633	-0.098	1.364
	Asthma				-		-	-		-	-		-
HIV Infected					-0.059	-0.248	0.129	-0.028	-0.218	0.161	-0.007	-0.196	0.183
Mental Health and Psychosocial Factors													
Depressive Symptoms (EPDS score)								-	-	-		-
Psychological Distress (SRQ-20)								-0.016	-0.035	0.002	-0.014	-0.033	0.005
IPV during pregnancy								-0.099	-0.251	0.052	-0.070	-0.222	0.082
Substance Use During Pregnancy & Primigravida													
Alcohol use (moderate-high risk)											-0.351**	-0.576	-0.127
Cotinine level													
	Non-smoker										-	-	-
	Passive										0.007	-0.171	0.185
	Active										-0.273*	-0.487	-0.059
Primigravida											-0.337***	-0.518	-0.157
			R2	p-value		R2	p-value		R2	p-value		R2	p-value
			0.024	0.001		0.051	<0.001		0.061	<0.001		0.096	<0.001
						Change R2	p-value		Change R2	p-value		Change R2	p-value
						0.027	0.001		0.010	0.046		0.035	<0.001

*p<0.05

**p<0.01

***p<0.001; 95% CI LL = 95% Confidence Interval Lower Limit, 95% CI UL = 95% Confidence Interval Upper Limit

#### Maternal predictors of head circumference adjusted for gestational age

In the final model (Block 4, Table 5), first time mothers were more likely to have infants with smaller adjusted head circumferences (B = -0.440, p = 0.007), as were mothers who used alcohol (B = -0.376, p = 0.011). Mothers diagnosed with gestational diabetes were more likely to have infants with larger age adjusted head circumferences (B = 1.386, p = 0.0021). In this hierarchical regression, the demographic variable block (R^2^ = 0.029, p<0.001), the mental health block (change in R^2^ = 0.016, p = 0.004), and the substance use block (change in R^2^ = 0.023, p<0.001) all significantly contributed to the explained variance in the final model but the pregnancy and physical health block did not (Table 5).

**Table 5:** Hierarchical regression predicting head circumference for gestational age (Z-score; N Final model = 888).

		Block 1			Block 2			Block 3			Block 4		
Variables		Coefficient	95% CI LL	95% CI UL	Coefficient	95% CI LL	95% CI UL	Coefficient	95% CI LL	95% CI UL	Coefficient	95% CI LL	95% CI UL
Demographics													
Clinic													
	Mbekweni	-	-	-	-	-	-	-	-	-	-	-	-
	TC Newman	-0.247*	-0.460	-0.034	-0.271*	-0.516	-0.026	-0.195	-0.452	0.062	-0.060	-0.345	0.225
Maternal age		0.032**	0.013	0.050	0.030**	0.009	0.051	0.036**	0.015	0.057	0.022	-0.001	0.046
Married or cohabitating		0.028	-0.185	0.240	0.050	-0.173	0.273	0.058	-0.167	0.283	-0.021	-0.253	0.210
SES													
	Low	-	-	-	-	-	-	-	-	-	-	-	-
	Low-moderate	-0.233	-0.505	0.040	-0.175	-0.457	0.108	-0.173	-0.457	0.110	-0.151	-0.438	0.135
	Moderate-high	-0.307*	-0.582	-0.032	-0.311*	-0.596	-0.025	-0.293*	-0.582	-0.004	-0.270	-0.564	0.024
	High	-0.040	-0.329	0.248	-0.055	-0.357	0.247	-0.101	-0.405	0.204	-0.107	-0.421	0.207
Food Insecure		-0.088	-0.305	0.130	-0.004	-0.234	0.226	0.030	-0.206	0.266	0.024	-0.215	0.262
Pregnancy and Physical Health													
BMI at Enrollment					0.003	-0.014	0.021	-0.003	-0.021	0.015	-0.009	-0.028	0.009
Pregnancy Complications													
	Hypertension				0.235	-0.309	0.778	0.320	-0.227	0.867	0.270	-0.298	0.838
	(Pre)-eclampsia				0.059	-0.612	0.731	0.031	-0.636	0.698	0.068	-0.602	0.738
	Anaemia				0.085	-0.160	0.331	0.070	-0.178	0.318	0.042	-0.205	0.290
	Gestational diabetes				1.107	-0.007	2.221	1.348*	0.168	2.527	1.386*	0.211	2.560
	Asthma				-	-	-	-	-	-	-	-	-
HIV Infected					-0.056	-0.335	0.222	0.002	-0.281	0.285	0.016	-0.269	0.302
Mental Health and Psychosocial Factors													
Depressive Symptoms (EPDS score)								-0.017	-0.040	0.007	-0.013	-0.036	0.010
Psychological Distress (SRQ-20)								-0.027	-0.059	0.005	-0.028	-0.060	0.004
IPV during pregnancy								-0.048	-0.276	0.181	-0.029	-0.260	0.201
Substance Use During Pregnancy & Primigravida													
Alcohol use (moderate-high risk)											-0.440*	-0.779	-0.101
Cotinine level													
	Non-smoker										-	-	-
	Passive										0.176	-0.093	0.444
	Active										-0.193	-0.515	0.130
Primigravida											-0.376**	-0.649	-0.103
			R2	p-value		R2	p-value		R2	p-value		R2	p-value
			0.029	<0.001		0.037	0.001		0.054	<0.001		0.077	<0.001
						Change R2	p-value		Change R2	p-value		Change R2	p-value
						0.009	0.351		0.016	0.004		0.023	<0.001

*p<0.05

**p<0.01

***p<0.001; 95% CI LL = 95% Confidence Interval Lower Limit, 95% CI UL = 95% Confidence Interval Upper Limit

## Discussion

This is the first African study to longitudinally, comprehensively measure maternal health including physical and mental disorder, and a range of exposures, and the association with birth outcomes. The study found high levels of poverty, food insecurity, HIV infection, IPV, and exposure to tobacco and alcohol, in mothers in a peri-urban African community in which there was good access to primary health care including antenatal, HIV prevention and obstetric care. Inclusion of a broad set of risk factors highlights significant physical and environmental risk factors as discussed but also emphasizes the role of psychosocial and substance abuse variables relating to critical birth outcomes. Approximately 17% of our sample was born prematurely which is higher than the estimated 12% for sub-Saharan Africa.[29] Almost all of the premature births were late premature (i.e. after 34 weeks). There are several factors prevalent in this population that may contribute to late prematurity including maternal HIV infection, smoking rates, pregnancy complications, or food insecurity. In particular, maternal smoking rates in this cohort are much higher than those reported for sub-Saharan Africa[30] and maternal HIV rates were also high.

Food insecurity is a strong predictor of gestational age, even when controlling for a number of related socioeconomic factors. Historically malnutrition has been a large consideration of antenatal programs and has often been addressed through food supplementation interventions. Women at both clinics had access to food supplementation programs, however, we still found high rates of food insecurity. Food insecurity was reported by 40% despite almost half receiving a social security grant. In our sample, food insecurity was significantly related to receiving a social security grant, such that those who received these grants were also more likely to report being food insecure compared to those who did not receive these grants (42% vs. 34%, p = 0.014). Improvement of the socioeconomic status of families will require more than social assistance or more substantial financial support, and may encompass strengthened educational programs and job creation initiatives. Future maternal child health interventions should focus on addressing the specific components of food insecurity including the psychological implications of worrying about having adequate access to food during the critical period of pregnancy.

Maternal pregnancy complications including hypertension, pre-eclampsia/eclampsia, and gestational diabetes, while uncommon in this cohort, were also strong predictors of gestational age; however, gestational diabetes was the only pregnancy complication that predicted weight adjusted for gestational age and head circumference adjusted for gestational age. Gestational diabetes as a strong predictor for poor birth outcomes is not surprising given the rich medical literature on this association; however, finding such a strong trend across two of the three outcomes given that only 1% of our sample screened for gestational diabetes underscores the importance of screening for gestational diabetes during pregnancy, particularly among populations that have increased risk factors, including high levels of overweight and obesity among women.[31] The low prevalence of these complications may reflect that mothers were young with a median age of around 26 years. The mothers in this cohort were also relatively healthy; the HIV infected mothers were nearly all on ART and had relatively well controlled HIV.

Mental health issues were common with a high prevalence of depression and psychological distress. These data confirm prior work in local and international contexts, which emphasize the public health importance of these conditions.[13] Among this sample of women, mental health and psychosocial factors did not significantly add to explained variance (change in R^2^), but in the context of a range of other variables were not strong predictors.[25, 32, 33] Conversely, substance use variable were strong predictors of birth weight and head circumference, particular moderate to high alcohol use risks. Our findings are consistent with the persistent link between intrauterine growth restriction and prenatal exposure to alcohol in the literature[34] and prenatal alcohol exposure can have long-term implications for child cognitive functioning and development.[20, 35] Furthermore, smoke exposure has been well described to adversely affect birth outcomes and long term child health including development of asthma, pneumonia or wheezing illness.[6] Reduction of alcohol consumption and smoking cessation programs should be a priority target pregnant women.[5]

### Strengths and limitations

Certain maternal physical and mental health factors during pregnancy may be too subtle to see in birth outcomes (gestational age, age adjusted birth weight, and age adjusted head circumference) but may impact later child developmental outcomes. For example, in these analyses HIV status did not significantly impact any of our main birth outcomes, however, there are concerns about subsequent impacts on child development.[36, 37] These impacts will be further examined as our cohort ages. Furthermore, the use of gestational age adjusted birth weights may have introduced a small amount of bias into the regression models. Additionally, due to study design (observational cohort) causality cannot be inferred.

The analyses presented here sometimes do not replicate previous findings from our cohort.[25] There are a number of reasons for this including differing sample sizes and differing measures for similar constructs. Furthermore, this analysis includes a more comprehensive range of variables, including maternal sociodemographic, food insecurity, physical health including pregnancy complications, mental health, and substance use factors. Controlling for a broader array of constructs is a strength of these analyses and allows us to disentangle the impacts of these factors on birth outcomes and better direct public health intervention development.

## Conclusions

In LMICs, child survival has been the major focus of health systems. However, with socio-economic development, better primary health care and availability of essential medicines, it is increasingly important to consider morbidity and development of chronic disease. This study has shown that despite a poor socioeconomic setting, and substantial maternal psychosocial adversity, the majority of infants were born normally at full term, with very few requiring hospitalisation after delivery and a very low mother-to-child transmission rate of HIV despite a high maternal HIV prevalence. This is a reflection of a good primary care antenatal and PMTCT programs and excellent cohort retention and follow-up. As primary health care is strengthened, so the importance of reducing exposures that adversely affect long term child health care is apparent. With the accumulating evidence of the importance of the first 1000 days in setting long term health, it is imperative that health systems address the broader determinants of child health and that preventative interventions are strengthened. Several modifiable risk factors including food insecurity, smoking, and alcohol consumption during pregnancy were identified as associated with negative birth outcomes, all of which are amenable to public health interventions. Such interventions must be urgently strengthened in LMIC settings.
